# A Liquid Level Measurement Technique Outside a Sealed Metal Container Based on Ultrasonic Impedance and Echo Energy

**DOI:** 10.3390/s17010185

**Published:** 2017-01-19

**Authors:** Bin Zhang, Yue-Juan Wei, Wen-Yi Liu, Yan-Jun Zhang, Zong Yao, Li-Hui Zhao, Ji-Jun Xiong

**Affiliations:** 1 Key Laboratory of Instrumentation Science & Dynamic Measurement, Ministry of Education, North University of China, Taiyuan 030051, China; zb0003@126.com (B.Z.); zhangyjnuc@hotmail.com (Y.-J.Z.); yaozong126@sina.com (Z.Y.); zhaolh6@gmail.com (L.-H.Z.); xiongjijunnuc@126.com (J.-J.X.); 2 Science and Technology on Electronic Test & Measurement Laboratory, North University of China, Taiyuan 030051, China; 3 Software School of North University of China, Taiyuan 030051, China; weiyuejuan@nuc.edu.cn

**Keywords:** ultrasonic sensor, ultrasonic impedance, echo energy, sealed metal container, liquid level measurement

## Abstract

The proposed method for measuring the liquid level focuses on the ultrasonic impedance and echo energy inside a metal wall, to which the sensor is attached directly, not on ultrasonic waves that penetrate the gas–liquid medium of a container. Firstly, by analyzing the sound field distribution characteristics of the sensor in a metal wall, this paper proposes the concept of an "energy circle" and discusses how to calculate echo energy under three different states in detail. Meanwhile, an ultrasonic transmitting and receiving circuit is designed to convert the echo energy inside the energy circle into its equivalent electric power. Secondly, in order to find the two critical states of the energy circle in the process of liquid level detection, a program is designed to help with calculating two critical positions automatically. Finally, the proposed method is evaluated through a series of experiments, and the experimental results indicate that the proposed method is effective and accurate in calibration of the liquid level outside a sealed metal container.

## 1. Introduction

The accurate measurement of the liquid level in a sealed metal container is essential to the production process and real-time control [1]. Especially in aviation, petroleum and chemical industries and other special areas of production, liquids in a metal sealed container mostly are volatile, flammable, explosive, and corrosive mixtures. At present, there are mainly four commonly used ultrasonic measurement methods [2,3].

The first types of methods are based on the principle of the sound speed. Their accuracy of liquid level measurement is affected by the change of sound velocity and the waveform of a received signal. The transit time is gained often after receiving the third or more periods of waveforms, which will affect the accuracy of the measurement. The measuring accuracy of this method is generally 0.25%–0.5% of its measuring range [4].

The second types of methods are penetrative methods. The most well-known product of this type is the famous PLI ultrasonic liquid level indicator produced by Class Instrumentation, Ltd. Sound waves must penetrate a liquid medium in a container and can be easily affected by internal impurities and bubbles in the liquid, which may lead to more errors. In addition, if the liquid medium is of poor transmittance or the diameter of the container is too large, then the echo signals may be very weak or even undetectable. Ultimately, it will make the measurement very difficult [5]. The accuracy of these methods is generally ±10 mm.

The third types of methods are noninvasive methods for measurement of the liquid level in a closed metal container, which can overcome the limitations of the two methods above. It is based on the use of ultrasonic Lamb waves propagating along the surface of a container wall. The characteristics of these Lamb waves would change substantially after contacting liquid. Such changes can be used as an indicator of liquid presence. However, the use of this method requires several strict conditions, as described in [6,7].

The fourth types of methods are the ultrasonic impedance methods, which are based on the difference in acoustic impedance between the liquid and gas medium. It measures the liquid level by comparing the echoes’ attenuation time [5]. However, multiple experiments and many values determined based on experience are required to calculate the attenuation time, making the result inaccurate and not flexible. In [8], the researchers achieved the liquid level by comparing the transmission coefficients of echoes when a sensor is above and below the liquid level, but its sensitivity was relatively low.

Given the strengths and weaknesses of these methods [5,6,7,8], this paper presents a method based on ultrasonic impedance and echo energy, of which the study focuses on detailed analysis and discussion of theory and experiment.

**Figure 1:**
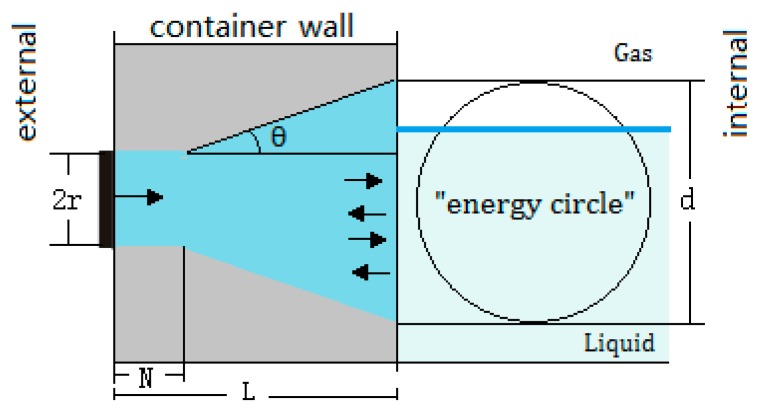
The principle of the proposed liquid level detection based on ultrasonic impedance and echo energy.

The proposed method is easy to operate and uses only one sensor to achieve an accurate liquid level measurement outside a sealed container. The liquid level is determined by echo energy inside the metal wall of a container, to which the sensor is directly attached. The propagation characteristics of acoustic waves that penetrate the gas–liquid medium of a container are not considered because it is easily affected by many factors, such as sound speed, impurities and bubbles in the liquid, or a container with a large diameter. Therefore, in this study, the concept of energy circle is proposed, based on which the model of liquid level measurement is established, and an algorithm is designed for calculating the echo energy dynamically to improve the detection accuracy. The principle of this proposed method is shown in Figure 1.

Table 1 includes the description of the symbols used in this paper.

## 2. Theory and Methods

### 2.1. Analysis of the Sound Field and Axial Response of the Sensor

**Figure 2:**
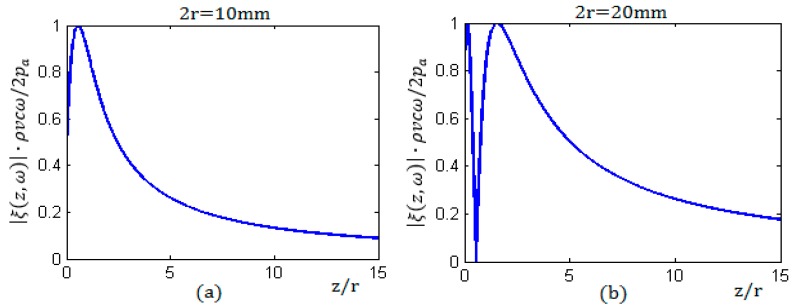
Axial responses of the sound pressure amplitude for the round piston sensors: (**a**) the sensor radius r = 5 mm; and (**b**) the sensor radius r = 10 mm.

According to the Schmerr model [9,10] and the analysis of the sound field by Roa-Prada [11,12], the sound field of the axial response of a round piston sensor in a solid medium consists of two parts, including the near-field region known as the Fresnel zone and the far-field region. The amplitude of sound pressure in the near field has many maxima and minima, and it will decrease as the distance increases in the far field [12], as shown in Figure 2. 

In the figures, *z* represents the distance to the center of a round sensor along the axial direction; the horizontal coordinate axis is represented by *z/r*, which is the ratio of the distance *z* and the sensor radius *r*. Figure 2a,b shows the characteristic of the sound pressure amplitude with the ratio *z/r*, the radius of sensor r equal to 5 mm and 10 mm, respectively. Because the sound intensity is proportional to the square of the amplitude, the sound intensity along the central axis has the same distribution characteristics.

The diffusion angle θ and the length of the near field *N* are calculated via Equations (1) and (2), respectively [13]:

(1) $$N=\frac{r^{2}}{\lambda_{c}},$$

(2) $$\theta =\arcsin\frac{1.22\lambda_{c}}{2r},$$

where $\lambda_{c}$ is the wavelength of the ultrasonic wave, and the geometrical meanings of other symbols are shown in Figure 1.

### 2.2. Calculation of Echo Energy

A metal wall of a container is regarded approximately as semi-infinite isotropic solid medium. When using a round piston sensor to transmit an ultrasonic beam in a certain incident frequency perpendicular to the outer surface of the container wall, as the beam propagates to the inner surface of the metal wall, the beam will be reflected and refracted, which follows the laws of reflection and refraction of sound waves [13]. The reflection coefficient *R* is determined by using Equation (3):

(3) $$R=\frac{\rho_{2}c_{2}\cos\theta_{i}-\rho_{1}c_{1}\cos\theta_{t}}{\rho_{2}c_{2}\cos\theta_{i}+\rho_{1}c_{1}\cos\theta_{t}},$$

where ρ is the medium density; c is the sound speed in the medium; $\theta_{i}$ is the incidence angle; and $\theta_{t}$ is the transmission angle.

Perpendicular to the outer surface of the wall, the sensor is excited to transmit an ultrasonic beam into the wall, which will be projected onto the inner surface of the wall and form a circular area, in which most of the energy is concentrated, whether the projection is in the near-field or in the far-field region. It depends on the relationship between the thickness of the wall (L) and the length of the near field (N). When L≤N, the projection is in the near field. Otherwise, the projection is in the far-field.

In this paper, we call the projected circular area as an "energy circle". Assuming that the diameter of an energy circle is denoted by *d*, and based on the diffusion angle θ which is calculated by Equation (2), the value of *d* can be obtained by using Equation (4):

(4) $$\{\begin{array}{c} d=2r\;\left(L\leq N\right)\\ d=2\left[r+\left(L-N\right)\tan\theta \right]\;(L>N) \end{array}.$$

In the actual liquid detection process, we adapt a single sensor with the function of transmitting and receiving ultrasonic waves. As soon as the sensor is excited by a momentary pulse and before the next excitation, the transmitting sensor is converted to a receiving sensor.

When the detection sensor moves from the bottom of a container to the top along the outer surface of the wall, the energy circle evolves in three different states, respectively. According to the propagation and attenuation characteristics of ultrasonic waves, we can use the following equations to calculate the echo energy for three states, respectively.

#### 2.2.1. The Energy Circle below or above the Liquid Level 

When the energy circle is below the interface of the liquid level, the total energy $P_{tl}$ received by the receiving sensor is obtained by Equation (5):

(5) $$P_{tl}=\overset{n}{\underset{i=1}{\sum }}I_{wi}\;\pi r^{2}=\overset{n}{\underset{i=1}{\sum }}\frac{{\left(P_{0}R_{ml}^{i}R_{ma}^{i-1}e^{-2i\alpha L}\right)}^{2}\pi r^{2}}{2Z_{m}}.$$

In the same way, when the energy circle is above the interface of the liquid level, the total energy $P_{tg}$ is calculated by Equation (6):

(6) $$P_{tg}=\overset{n}{\underset{i=1}{\sum }}I_{wi}\;\pi r^{2}=\overset{n}{\underset{i=1}{\sum }}\frac{{\left(P_{0}R_{mg}^{i}R_{ma}^{i-1}e^{-2i\alpha L}\right)}^{2}\pi r^{2}}{2Z_{m}}.$$

The metal container and the gas–liquid medium are fixed for a given testing environment. If the incident frequency is taken as a constant value, then the sound pressure $P_{0}$ is constant, and the values of $P_{tg}$ and $P_{tl}$ received by the receiving sensor will also be constant.

#### 2.2.2. Two Parts of the Energy Circle Divided by the Liquid Level

**Figure 3:**
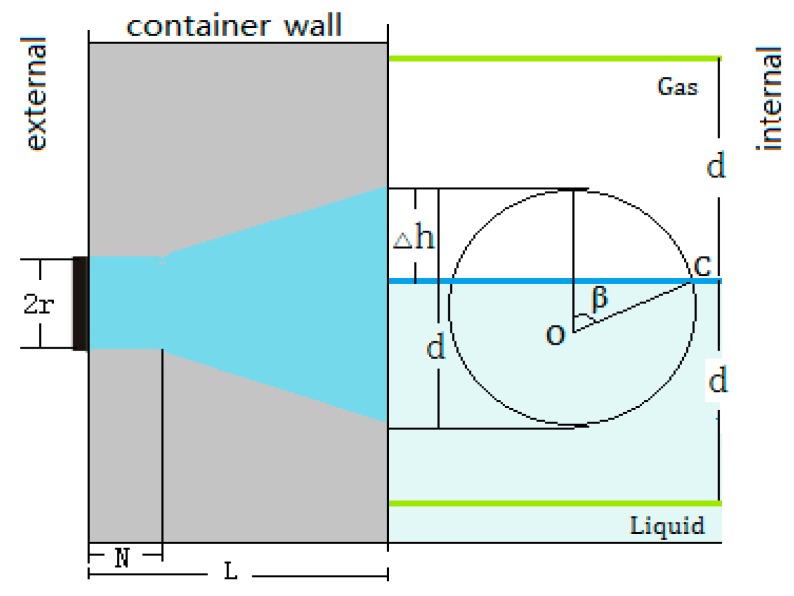
The two parts of the energy circle divided by the liquid level.

In this state, the energy circle is divided into two parts by the liquid level, as shown in Figure 3.

Assuming that the total area of the energy circle is $A_{e}$, when the top of the energy circle exceeds the liquid level, the height is represented by Δh and 0≤Δh≤d; the area of the energy circle above the liquid level is denoted by $A_{1}$. Let $r_{A}=A_{1}/A_{e}$. 

Considering the whole energy circle in two cases, the total energy $P_{t}$ received by the receiving sensor should be the superposition of the two parts of the energy circle and can be determined by Equation (7):

(7) $$P_{t}=P_{tg}+P_{tl}=\overset{n}{\underset{i=1}{\sum }}\frac{{\left(P_{0}R_{ma}^{i-1}e^{-2i\alpha L}\right)}^{2}r^{2}\left[R_{ml}^{2i}(1-r_{A}\right)+R_{mg}^{2i}r_{A}]}{2Z_{m}}.$$

#### 2.2.3. The Critical State

If $r_{A}$ is viewed as a critical value of 0 or 1, Equation (7) will become Equation (5) or Equation (6), respectively. Therefore, Equations (5) and (6) are the two critical states in which an energy circle is just in the state above or below the liquid level, respectively.

Through the above analysis, when the sensor moves up gradually along the outer surface of a container from the location below the liquid level, the total energy $P_{t}$ received by the receiving sensor in three different states are determined by Equations (5)–(7), respectively.

#### 2.2.4. Calibration of a Liquid Level

From Figure 3, the values of the height Δh and the ratio $r_{A}$ can be obtained by using Equations (8) and (9), respectively, where 0≤β≤π. Its meaning is shown in Figure 3:

(8) $$\Delta h=\frac{d}{2}\left(1-cos\;\beta \right),$$

(9) $$r_{A}=\frac{1}{\pi }\left(\beta -sin\;\beta \;cos\;\beta \right).$$

When the metal wall and the internal gas–liquid medium in a container and other initial conditions are fixed, Equation (7) can be rewritten as Equation (10):

(10) $$P_{t}=C_{1}r_{A}+C_{2.}$$

**Figure 4:**
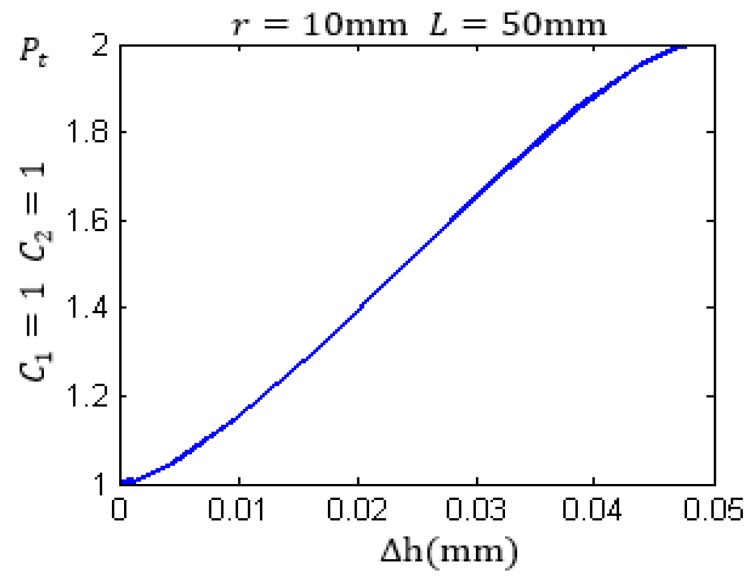
The relationship between the total energy $P_{t}$ and Δh.

Since acoustic impedance is quite different between the liquid medium and the gaseous medium, and, generally, $Z_{l}\gg Z_{g}$, there is the following relationship between them:

(11) $$\frac{R_{mg}}{R_{ml}}=\frac{\rho_{m}c_{m}-\rho_{g}c_{g}}{\rho_{m}c_{m}+\rho_{g}c_{g}}\cdot \frac{\rho_{m}c_{m}+\rho_{l}c_{l}}{\rho_{m}c_{m}-\rho_{l}c_{l}}>\frac{\rho_{m}c_{m}-\rho_{g}c_{g}}{\rho_{m}c_{m}+\rho_{l}c_{l}}\cdot \frac{\rho_{m}c_{m}+\rho_{l}c_{l}}{\rho_{m}c_{m}-\rho_{g}c_{g}}=1,$$

where $C_{1}$ and $C_{2}$ are constant values for a given detection. Based on Equation (7) and Equation (11), $C_{1}>0$ and $C_{2}>0$. Therefore, with the increase of Δh from 0 to *d*, the energy circle is moved from the state below the liquid level to the state above the liquid level. In this process, the relationship between the total energy $P_{t}$ received by the receiving sensor and the ratio $r_{A}$ is linear. According to Equations (8)–(10), the diagram of the relationship between the total energy $P_{t}$ and the Δh can be determined. Figure 4 shows this relationship with $C_{1}=1,\;C_{2}=1$, *r* = 10 mm, and *L* = 50 mm.

**Figure 5:**
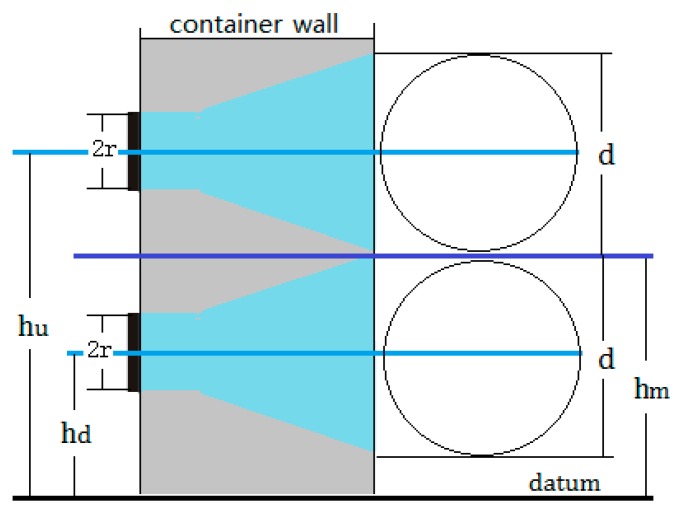
Determination of two critical positions of the energy circle and calibration of liquid level.

Then, we can use this relationship to determine the exact position of the measured liquid level $h_{m}$. If we can find the two critical heights of $h_{d}$ and $h_{u}$ that correspond to the two critical states described in Section 2.2.3, the measured liquid level $h_{m}$ will be determined by Equation (12), and their geometric description is shown in Figure 5:

(12) $$h_{m}=\frac{h_{d}+h_{u}}{2}.$$

In the actual process, when the two critical positions are found, the values of $h_{d}$ and $h_{u}$ are obtained through external scale values of the container, or via the use of the infrared distance measuring instruments.

## 3. Experimental Results

### 3.1. Configuration of Experimental Environment and Initial Conditions

In the evaluation of the proposed method, we used a series of alloy sealed metal containers with different wall thickness, liquid media, and gaseous media, which contain water and air. 

The temperature of the test environment is −10–80 °C. According to the detection environment and conditions, there are *Z_m_* = 17 × 10^6^ kg/m^2^·s, *Z_g_* = 0.0004 × 10^6^ kg/m^2^·s, *Z_l_* = 1.48 × 10^6^ kg/m^2^·s, $R_{mg}=R_{ma}=0.9999529$, and $R_{ml}=0.8398268$.

Considering the propagation characteristics of ultrasonic waves in a metal wall, we selected two kinds of sensors whose center frequency $f_{c}$ was 1 MHz and diameters were 10 mm and 20 mm, respectively. Excitation voltage U used in the experiment was 225 V. The repetition frequency of excitation pulse $f_{r}$ was 100 Hz, with a repetition period T of 0.01 s. Then, we take *L* = 50 mm, as an example, to discuss the main detection process.

**Figure 6:**
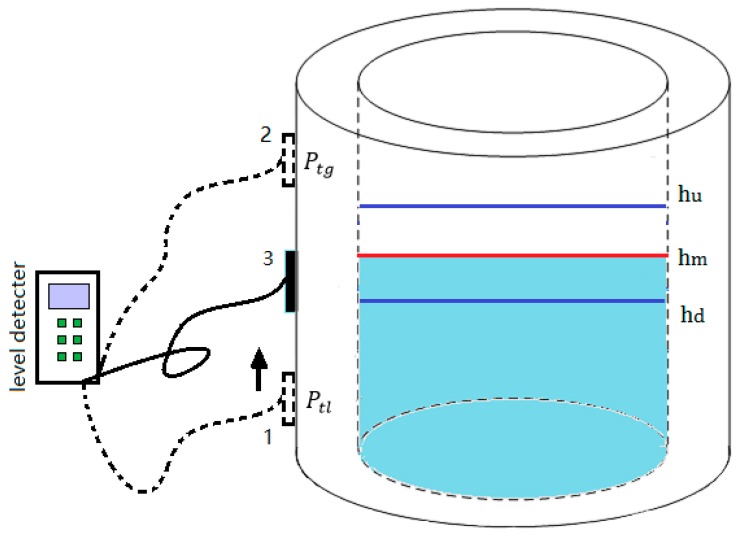
The operating schematic diagram of liquid level measurement in the experiment.

In the experiments, the measurement process can be divided into the following three steps. The diagram of operation is shown in Figure 6.

The first step is to find the critical position $h_{d}$ below. The sensor is moved from position 1 to the top of the container slowly along the surface of the container wall to keep good coupling. The value of echo energy does not change until the sensor is moved to the critical position $h_{d}$ below the liquid level. At that time, the buzzer in the circuit will be triggered and beep, and then the scale value of $h_{d}$ can be read.

The second step is to find the above critical position $h_{u}$. Continue to move the sensor from position $h_{d}$ to the top of the container. In this process, the buzzer will keep beeping until the echo energy value does not change, which means that the sensor reaches the critical position above the liquid level, and the scale value of $h_{u}$ can be obtained.

In the third step, the liquid level $h_{m}$ is measured by substituting $h_{d}$ and $h_{u}$ into Equation (12). In order to obtain more accurate results, these steps are recommended to repeat more than once.

### 3.2. The Results of the Experiment

**Figure 7:**
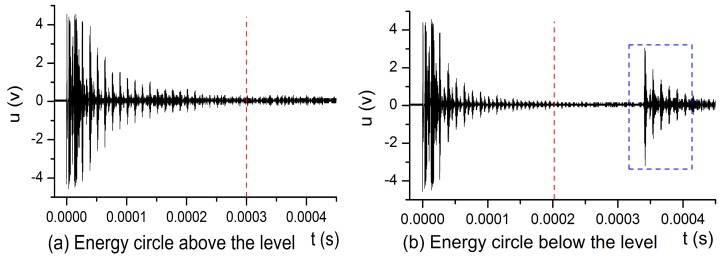
(**a**) The energy circle is above the liquid level; and (**b**) the energy circle is below the liquid level, with the thickness of a metal wall being 50 mm.

The echo signals shown in Figure 7 are the features of the two states that the energy circle was located above and below the liquid level, respectively, within a transmission period of 0.01 s. 

In Figure 7, the signals in the dashed blue box are the echo signals that the transmitted ultrasonic beam is reflected when passing through a gas or a liquid medium by the opposite inner surface of the metal container wall, which is used to detect the liquid level by the fourth type of methods mentioned in the Introduction. However, in this study, these echo signals are not adapted.

From Figure 7, we can see that the echo signals in the metal wall disappeared after the time of t=0.0002 s when the energy circle was below the liquid level, and, after t=0.0003 s, the echo signals in the metal wall also disappeared when the energy circle was above the liquid level. About t=0.00034 s later, the signals penetrating the gaseous or liquid medium were reflected back from the opposite wall of the metal container.

Through the above analysis, the time t is a crucial element to calculate echo energy. In this research, we designed an algorithm to dynamically obtain its value, which will be introduced next. This process is automatically calculated in the program.

**Figure 8:**
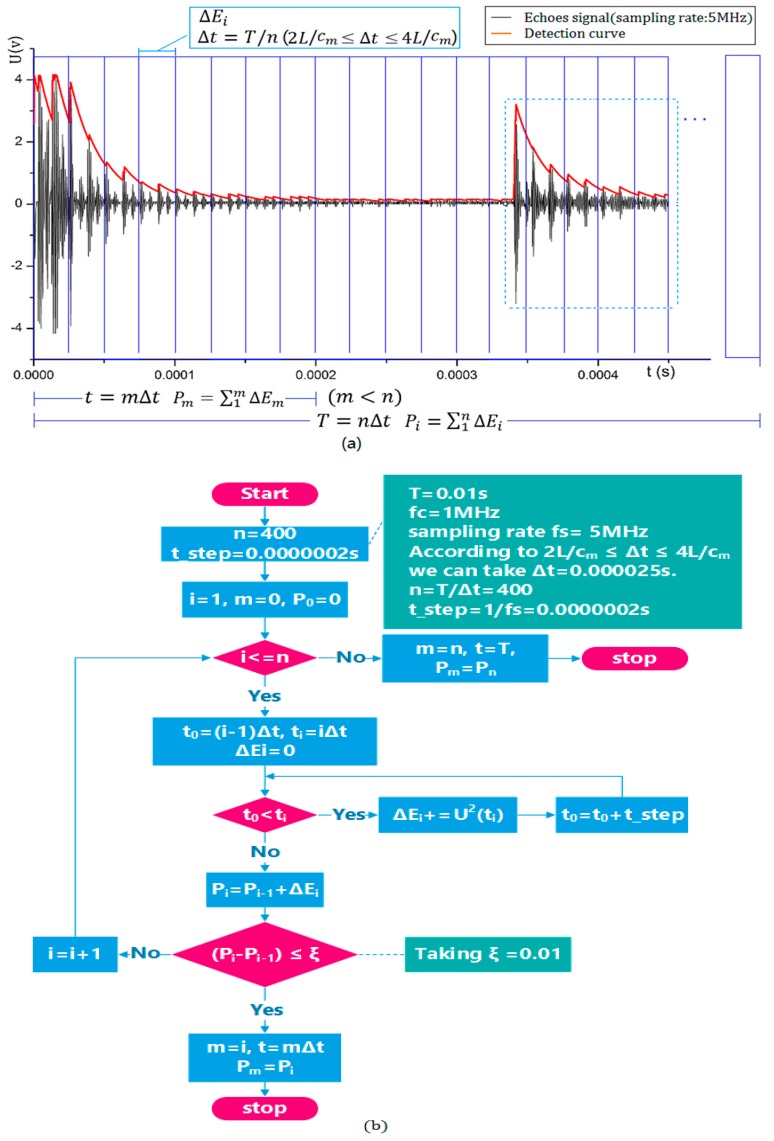
The algorithm that dynamically calculates the value of the time *t* and the valid echo energy: (**a**) is a schematic diagram, and (**b**) is a flow chart.

The algorithm for determining the value of *t* and the valid echo energy dynamically is shown in Figure 8. An echoes waveform is obtained by 5 MHz sampling of the original echoes; the red line is the envelope detection curve. It can be seen that the detection curve is convergent due to the attenuation of echo energy, whether the echoes are generated at the first inner surface or at an opposite surface. Therefore, we can use the feature of detection curve to obtain the value of t.

The detection curve can be denoted by the function $y_{t}=U\left(t\right)$. We assume that the total energy is $P_{i}=\sum_{1}^{n}\Delta E_{i}\;\left(0\leq i\leq n\right)$ in a repetition period T, and let Δt=T/n, (where $2L/c_{m}\leq \Delta t\leq 4L/c_{m}$). Then, $\Delta E_{i}$ can be calculated by $\Delta E_{i}=\sum_{\left(i-1\right)\Delta t}^{i\Delta t}{y_{t}}^{2}$. Finally, we can obtain the value of total energy $P_{n}$ when t=T. 

However, in actual detection, in order to make the difference between the two parts of echo energy more salient as the energy circle is divided by the liquid level, we need to obtain $P_{m}=\sum_{1}^{m}\Delta E_{m}$, where m<n and t=mΔt. It means that we do not want to get all echo energy values and do not care about the echoes at the opposite surface in the blue dashed box. 

Then, before calculating the value of $P_{m}$, the value of m must be obtained first. Therefore, the value of time t is converted to the value of *m*. If the function $y_{t}=U\left(t\right)$ is convergent before the opposite echo arrives, we can take $\Delta P=P_{i}-P_{i-1}$, when $\underset{i}{lim}\Delta P=\to 0$, take m=i, and obtain t=m⋅Δt.

On the other hand, if the function is not convergent before the opposite echoes arrive, it implies that the echoes generated from two interfaces are partially overlapped because of a very small diameter of the container. In this case, the value of the time t would be determined by the attenuation of opposite echoes, which is likely to make a small difference depending on the material of the container and the internal gas–liquid medium. 

**Figure 9:**
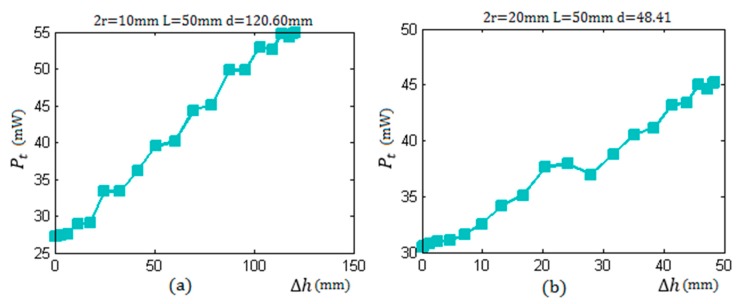
The change law of the actual total energy $P_{t}$ received by the sensor with the increase of Δh from 0 to d with the thickness of a container wall being 50 mm: (**a**) the sensor radius r = 5 mm; and (**b**) the sensor radius r = 10 mm.

Figure 9 shows the actual measurement results with the container thickness being 50 mm. The values of the mechanical vibration energy of ultrasonic waves are converted to the values of the actual electric power by the receiving circuit. Comparing the results shown in Figure 4 and Figure 9, it can be seen that the change of the echo energy received by the receiving sensor is consistent with the change law of the curve of the theoretical values.

Table 2 presents the average values of the result of taking measurements three times. The symbol $h_{l}$ represents the actual height of liquid level in a container, $\bar{h_{m}}$ is the average measuring result of the proposed method, and $\bar{\Delta E}$ is the average error. The values of $\bar{h_{p}}$ are the average results measured by PLI indicator whose average error is represented by $\bar{\Delta E_{p}}$. Obviously, in the same test environment, the error $\bar{\Delta E_{p}}$ is roughly between 4 and 9 mm, in line with its accuracy range of ±10 mm. It is higher than the error $\bar{\Delta E}$, which is less than 4 mm.

**Figure 10:**
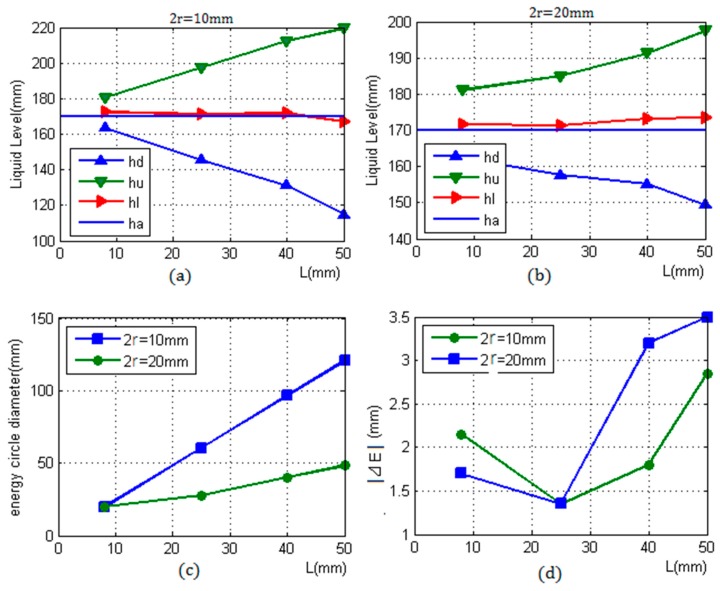
Two detection results of different sensors: (**a**) 2r = 10 mm; (**b**) 2r = 20 mm; (**c**) comparison of energy circle diameters for two kinds of sensors; and (**d**) errors.

Table 2 and Figure 10a–c show that, when the diameter of the sensor increases, the ultrasonic beam will become more concentrated; the divergence angle will become smaller; the near-field length will become longer; the interval between two critical positions will become smaller; the sensitivity will become higher and the resolution will become lower; and vice versa.

Figure 10d demonstrates that the error of the smaller diameter sensor is higher than that of the larger diameter sensor when the wall thickness is less than 25 mm. However, as the thickness of the container increases, the error of the smaller diameter sensor will be smaller than that of the larger diameter sensor. 

In Figure 10a,b, the measured liquid level $h_{m}$ is calculated by the two critical positions $h_{d}$ and $h_{u}$. The accuracy of the measurement result will depend on the two critical positions found by the change feature of echo energy. In the detection process, the value of echo energy will change, as described in Equation (7). The slope item $\sum R_{mg}^{2i}-R_{ml}^{2i}$ determines the urgency of this change. Therefore, the larger the difference between the reflection coefficients $R_{mg}$ and $R_{ml}$, the more obvious the change and the higher the measurement accuracy, which is determined by the gas–liquid medium in a container.

## 4. Discussion

Compared with other methods, the proposed ultrasonic method for liquid level measurement is more effective as other methods cannot meet the requirement of the detection conditions. The proposed method does not need to be installed in advance and does not damage the physical structure and the integrity of containers. It also does not require the ultrasonic beam to penetrate the liquid medium in containers or containers with a large diameter. Therefore, the proposed method is more secure, convenient and flexible, although it maybe not achieve higher accuracy than other methods. 

In addition, this method also has its disadvantages. There are two uncertain factors affecting the measurement result. One is the coupling matching layer that affects the stability of the energy of incident waves. Since the proposed method is based on the change of echo energy in a container wall, the premise is that the energy of incident acoustic waves should remain constant when the sensor moves to different positions during a detection process. However, it is not easy to control it in the actual operation, which depends on the roughness of the container surface, and the coupling matching layer often needs to be adjusted repeatedly to obtain a satisfactory result. The other uncertainty is the difference of ultrasonic impedance between gas and liquid in containers. If this difference is so small that two parts of echo energy are quite similar to each other, as the sensor is, respectively, above and below the liquid level, and the measurement will not be possible.

## 5. Conclusions

The experimental results demonstrate that the proposed method is effective for liquid level measurement outside a sealed container. The proposed method has the advantages in non-intrusive measurement and a wider range of applications. The selection of the center frequency of a sensor is important in this approach, which affects the magnitude of echo energy in the wall. In the actual detection process, according to the material of a container and the characteristics of the sensor used, a selection program may be designed to determine the optimal detection frequency. Furthermore, more than one sensor can be chosen to detect the same level, and the average value of sensor measurements can be used as a final result to improve the accuracy of the measurement.

## Figures and Tables

**Table 1:** The symbols used in this paper and their meanings.

Symbol	Meaning
r	the radius of a sensor
θ	the diffusion angle
α	the attenuation coefficient
$f_{c}$	the center frequency of a sensor
L	the thickness of a container wall
N	the length of the near field
$\lambda_{c}$	the wavelength of compressional wave in a metal wall
d	the diameter of the energy circle
ρ	the density of a medium
c	the wave speed in a medium
$Z_{l}$	the acoustic impedance of a liquid medium $Z_{l}=\rho_{l}c_{l}$
$Z_{g}$	the acoustic impedance of a gaseous medium $Z_{g}=\rho_{g}c_{g}$
$Z_{m}$	the acoustic impedance of a metal container wall $Z_{m}=\rho_{m}c_{m}$
$R_{mg}$	the reflection coefficient at the surface between wall and gaseous medium
$R_{ml}$	the reflection coefficient at the surface between wall and liquid medium
$R_{ma}$	the reflection coefficient at the surface between wall and gas medium
m,g,l,a	the subscripts associated with a metal, a gas, a liquid and an air medium
U	the excitation voltage
$f_{r}$	the repetition frequency of a pulse
T	the repetition period
$P_{0}$	the incident sound pressure
$I_{w}$	the sound intensity at outer surface of a wall
$P_{t}$	the total energy received by a sensor as the energy circle is divided by the liquid level
$P_{tg}$	the total energy received by a sensor as the energy circle is above the liquid level
$P_{tl}$	the total energy received by a sensor as the energy circle is below the liquid level
$h_{l}$	the actual height of the liquid level
$h_{u}$, $\bar{h_{u}}$	the critical height above the liquid level and its average value
$h_{d}$, $\bar{h_{d}}$	the critical height below the liquid level and its average value
$h_{m}$, $\bar{h_{m}}$	the measured height of the liquid level and its average value

**Table 2:** The result of measurements using two different diameters of sensors and PLI indicator (mm).

L	2r	N	d	$\bar{h_{d}}$	$\bar{h_{u}}$	$\bar{h_{m}}$	$h_{l}$	$\bar{\Delta E}$	$\bar{h_{p}}$	$\bar{\Delta E_{p}}$
8	10	4	19.68	163.6	180.7	172.15	170	2.15	176.61	6.61
25	10	4	60.50	145.4	197.3	171.35	170	1.35	175.32	5.32
40	10	4	96.58	131.1	212.5	171.8	170	1.8	163.95	−6.05
50	10	4	120.60	114.9	219.4	167.15	170	−2.85	161.1	−8.9
8	20	15.9	20.00	162.3	181.1	171.7	170	1.7	176.53	6.53
25	20	15.9	27.59	157.7	185	171.35	170	1.35	174.25	4.25
40	20	15.9	40.08	155.1	191.3	173.2	170	3.2	163.32	−6.68
50	20	15.9	48.41	149.4	197.6	173.5	170	3.5	161.9	−8.1
